# Supplementary material for: Heat adaptation of phage T7 under an extended genetic code
Source: Virus Evol. 2021 Dec 1;7(2):veab100. doi: 10.1093/ve/veab100 (PMC8923235; doi:10.1093/ve/veab100)
Supplement: veab100_Supp [file veab100_supp.zip › Sequencing_Analysis.zip!/Sequencing_Analysis/A1/evidence/JC_SIDE_1_60.html]

BRESEQ :: Evidence


| New junction evidence | | | | | | | | | | | |
| --- | --- | --- | --- | --- | --- | --- | --- | --- | --- | --- | --- |
|  | | seq id | position | reads (cov) | reads (cov) | score | skew | freq | annotation | gene | product |
| \* | ? | NC\_001604.1 | = 13568 | 84 (0.030) | 12 (0.000) | 7/586 | 43.0 | 8.7% | intergenic (–/–) | *–/–* | –/– |
|  | ? | NC\_001604.1 | 14339 = | 169 (0.060) |  |  |  |  | intergenic (–/–) | *–/–* | –/– |
| Rejected: Coverage evenness skew score above cutoff. | | | | | | | | | | | |
| Rejected: Frequency below/above cutoff threshold. | | | | | | | | | | | |

Only 100 of 2355 total aligned reads displayed.  

`GGATTCTTGATGACTTTCCAGACGACTACGAGAAGTTTCGCTGGAGAGTCCCATTCTAATACGACTCACTAAAGGAGACACACCATGTTCAAACTGATTAAGAAGTTAGGCCAACTGCTGGTTCGTATGTACAACGTGGAAGCCAAGCGACTGAACGATGAGGCTCGTAAAGAGGCCACACAGTCACGCGCTCTGGCGATTCGCTCCAACGAACTGGCTGACAGTGCATCCACTAAAGTTACCGAGGCTGCCCGTGTGGCAAACCAAGCTCAACAGCTTTCCAAATTCTTTGAGTAATCAAACAGGAGAAACCATTATGTCTAACGTAGCTGAAACTATCCGTCTATCCGATACAGCTGACCAGTGGAACCGTCGAGTCCACATCAACGTTCGCAACGGTAAGGCGACTATGGTTTACCGCTGGAAGGACTCTAAGTCCTCTAAGAATCACACTCAGCGTATGACGTTGACAGATGAGCAAGCACTGCGTCTGGTCAATGCGCTTACCAAAGCTGCCGTGACAGCAATTCATGAAGCTGGTCGCGTCAATGAAGCTATGGCTATCCTCGACAAGATTGATAACTAAGAGTGGTA  >  NC_001604.1/13268‑13861`  

                                                                                                                                                                                                                                                                                                            `|`                                                                                                                                                                                                                                                                                                       
  `agtgagtcgtattagaatgggactctccagaAGTTTCGCTGGAGAGTCCCATTCTAATACGACTCACTAAAGGAGACACACCATGTTCAAACTGATTAAGAAGTTAGGCCAACTGCTGGTTCGTATGTACAACGTGGAAGCCAAGCGACTGAACGATGAGGCTCGTAAAGGGGCCACACAGTCACGCGCTCTGGCGATTCGCTCCAACGAACTGGCTGACAGTGCATCCACTAAAGTTACCGAGGCTGCCCGTGTGGCAAACCAAGCTCAAAAGCTTTCCAAATTCTTTTAGTAATCaaaa                                                                                                                                                                                                                                                                                                     >  1:201741/29‑300 (MQ=255)`  
    `tCTTGATGCCTTTCCATACGACTCCGAGAAGTTTCGCTGGAGAGTCCCATTCTAATACGACTCACTAAAGGAGACACCCCATGTTCAAACTGATTAAGAAGTTAGGCCAACTGCTGGTTCGTATGTACAACGTGGAAGCCAAGCGACTGAACGATGAGGCTCGTAAAGAGGCCACACAGTCACGCGCTCTGGCGATTCGCTCCAACGAACTGGCTGACAGTGCATCCACTAAAGTTACCGAGGCTGCCCGTGTGGCAAACCAAGCTCAACAGCTTTCCAAATTCTTTGAGTAATCaatagg                                                                                                                                                                                                                                                                                                   <  1:209929/301‑5 (MQ=255)`  
    `tCTTGATGACTTTCCAGACGACTACGGGAAGTTTCGCTGGAGAGTCCCATTCTAATACGACTCACTAAAGGAGACACACCATGTTCAAACTGATTAAGAAGTTAGGCCAACTGCTGGTTCGTATGTACAACGTGGAAGCCAAGCGACTGAACGATGAGGCTCGTAAAGAGGCCACACAGTCACGCGCTCTGGCGATTCGCTCCAACGAACTGGCTGACAGTGCATCCACTAAAGTTACCGAGGCTGCCCGTGTGGCAAACCAAGCTCAACAGCTTTCCAAATTCTTTGAGTAATCAAACAg                                                                                                                                                                                                                                                                                                   <  1:421245/301‑1 (MQ=255)`  
     `cTTGATGACTTTCCAGACGACTACGAGAAGTTTCGCTGGAGAGTCCCATACTAATACGACTCACTAAAGGAGACACACCATGTTCAAACTGATTAAGAAGTTAGGCCAACTGCTGGTTCGTATGTACAACGTGGAAGCCAAGCGACTGAACGATGAGGCTCGTAAAGAGGCCACACAGTAACGCGCTCTGGCGATTCGCTCCAACGAACTGGCTGACAGTGCATCCACTAAAGTTACCGAGGATGCCCGTGTGGCAAACCAAGCTCAACAGCTTTCCAAATTCTTTGAGTAATCAAACAgg                                                                                                                                                                                                                                                                                                  >  1:406627/1‑301 (MQ=255)`  
          `tgaCTTTCCAGACGACTACGAGAAGTTTCGCTGGAGAGTCCCATTCTAATACGACTCACTAAAGGCGACACACCATGTTCAAACTGATTAAGAAGTTAGGCCAACTGCTGGTTCGTATGTACAACGTGGAAGCCAAGCGACTGAACGATGAGTCTCGTAAAGATGCCACACAGTCACGCGCTCTGGCGATTCGCTCCAACGAACTGGCTGACAGTGCATCCACTAAAGTTACCGAGGCTGCCCGTGTGGCAACCCAAGCTCAACAGCTTTCCAACTTCTTTGATTAATCAAACAGGAaaaa                                                                                                                                                                                                                                                                                             >  1:177893/1‑301 (MQ=255)`  
            `catatCCAGACGACTACGAGAAGTGTCGCTGGAGAGTCCCATTTTAATCCGATTCACTAAAGGAGACACACCATGTTCAAACTGATTAAGAAGTTAGGCCAACTGCTGGTTCGTATGTACAACGTGGAAGCCAAGCGACTGAACGATGAGGCTCGTAAAGAGGCCACACAGTCACGCGCTCTGGCGATTCGCTCCAACGAACTGGCGGACAGTGCATCCACTAAAGTTACCGAGGCTGCCCGTTTGGCAAACCAAGCTCAACAGCTTTCCAAATTCTTTGAGTAATCAAACAGGAGAAAcc                                                                                                                                                                                                                                                                                           <  1:1371/297‑1 (MQ=255)`  
                              `agaAGTTTCGCTGGAGAGTCCCATTCTAATACGACTCACTAAAGGAGACACACCATGTTAAAACTGATTAAGAAGTTATGCCAACTGCTGGTTCGTATGTACAACGTGGAAGCCAAGCTACTGAACGATGAGGCTCGTAAAGAGGCCACACAGTCACGCGCTCTGGCGATTCGCTCCAACGAACTGGCTGACAGTGCATCCACTAAAGTTACCGAGGCTGCCCGTGTGGCAAACCAAGCTCAACAGCTTTCCAAATTCTTTGAGTAATCAAACAGGAGAAATCAATATGatcgtttctgac                                                                                                                                                                                                                                                                         <  1:487291‑M1/301‑13 (MQ=255)`  
                                      `cGCTGGAGAGTCCCATTCTAATACGACTCACTAAAGGAGACACACCATGTTCAAACTGATTAAGAAGTTAGGCCAACTGCTGGTTCGTATGTACAACGTGGAAGCCAAGCGACTGAACGATGAGGCTCGTAAAGAGGCCACACAGTCACGCGCTCTGGCCATTCGCTCCAACGAACTGGCTGACAGTGCATCCACTAAAGTTACCGAGGCTGCCCGTGTGGCAAACCAATCTCAACAGCTTTCCAAATGCTTTGATTAATCAACCAGGACAAACCATAATGatcttttctgacatcgaagc                                                                                                                                                                                                                                                                 >  1:258511‑M1/1‑281 (MQ=255)`  
                                       `gCTGGAGAGTCCCATTCTAATACGACTCACTAAAGGATACACACCATGTTCAAACTGATTAAGAAGTTAGGCCAACTTCTGGTTCGTATGTACAACGTGGAATCCAAGCGACTGAACGATGAGGCTCGTAAAGACGCCACACAGTTAAGCGATCTGGCGATTCGTTCCAACGTACTGGCTGACAGTGCATCCACTAAAGTTACCGAGGCATCCCGTGTGGCAAACTAAGATCAACAGCTTTCCAAATTCTTTGAGTAATCAAACAAGAGAAACCATTATGattgtttctgacatcgaagct                                                                                                                                                                                                                                                                >  1:424976‑M1/1‑280 (MQ=255)`  
                                          `ggAGAGTCCCATTCTAATACGACTCACTAAAGGAGACACACCATGTTCAAACTGATTAAGAAGTTAGGCCAACTGCTGGTTCGTATGTACAACGTGGAAGCCAAGCGACTGAACGATGAGGCTCGTAAAGAGGCCACACAGTCACGCGCTCTGGCGATTCGCTCCAACGAACTGGCTGACAGTGCATCCACTAAAGTTACCGAGGCTGCCCGTGTGGCAAACCAAGCTCAACAGCTTTCCAAATTCTTTGAGTAATCAAACAGGAGAAACCATTATGatagtttctgacatcgaagctaac                                                                                                                                                                                                                                                             >  1:445505‑M1/1‑277 (MQ=255)`  
                                           `aaagagaaagttataaaacgactcactAAAGGAGACACACCATGTTCAAACTGATGAAAAAGTTAGGCCAACTGCTGGTTCGTATGAAAAACGTGGAAGCCAAGCGACTGAACGATAAGGCTCGTAAAGAGGCCACACAGTCACGCGCTCTGGCGATTCGCTCCAACGAACTGGCTGACAGTGCATCCACTAAAGTTACCGAGGCTGCCCGTGTGGCAAACCAAGCTCAACAGCTTTCCAAATTCTTTGAGTAATCAAACAGGAGAAACCATTATGatcgtttctgacatcgaagctaacg                                                                                                                                                                                                                                                            <  1:43382‑M1/288‑26 (MQ=255)`  
                                               `gTCCCATTCTAATACGACTCACTAAAGGAGACACACCATGTTCAAACTGATTAAGAAGTTAGGTCAACTGCTGGTTCGTATGTACAACGTGGAAGCCAAGCGACTGAACGATGAGGCTCGTAAAGAGGCCACACAGTCACGCGCTCTGGCGATTCGCTCCAACGAACTGGCTGACAGTGCATCCACTAAAGTTACCGAGGCTGCCCGTGTGGCAAACCAAGCTCAACAGCTTTCCAAATTCTTTGAGTAATCAAACAGGAGAAACCATTATGatcgtttctgacatcgaagctaacgcccc                                                                                                                                                                                                                                                        >  1:218791‑M1/1‑272 (MQ=255)`  
                                                     `ttCTAATACGACTCACTAAAGGAGACACACCATGTTCAAACTGATTAAGAAGTTAGGCCAACTGCTGGTTCGTATGTACAACGTGGAAGCCAAGCGACTGAACGATGAGGCTCGTAAAGAGGCCACACAGTCACGCGCTCTGGCGATTCGCTCCAACGAACTGGCTGACAGTGCATCCACTAAAGTTACCGAGGCTGCCCGTGTGGCAAACCAAGCTCAACAGCTTTCCAAATTCTTTGAGTAATCAAACAGGAGAACCCATTATGatcgattctgacatcgaagctaacgcccggtctct                                                                                                                                                                                                                                                  >  1:307361‑M1/1‑266 (MQ=255)`  
                                                     `ttCTAATACGACTCACTAAAGGAGACACACCATGTTCAAACTGATTAAGAAGTTAGGCCAACTGCTGGTTCGTATGTACAACGTGGAAGCCAAGCGACTGAACGATGAGGCTCGTAAAGAGGCCACACAGTCACGCGCTCTGGCGATTCGCTCCAACGAACTGGCTGACAGTGCATCCACTAAAGTTACCGAGGCTGCCCGTGTGGCAAACCAAGCTCAACAGCTTTCCAAATTCTTTGAGTAATCAAACAGGAGAAACCATTATGatcgtttctgacatagaagctaactcactcttaga                                                                                                                                                                                                                                                  >  1:206367‑M1/1‑266 (MQ=255)`  
                                                     `ttCTAATACGACTCACTAAAGGAGACACACCATGTTCAAACTGATTAAGAAGTTAGGCCAACTGCTGGTTCGTATGTACAACGTGGAAGCCAAGCGACTGAACGATGAGGCTCGTAAAGAGGCAACACAGTCACGCGCTCTGGCGATTCGCTCCAACGAACTAGCTGACAGTGCATCCACTAAAGTTACCGAGGCTGCCCGTGTGGCAAACCAAGCTCAACAGCTTTCCAAATTCTTTGAGTAATCAAACAGGAGAAACCATTATGatcgtttctgacatcgcagctaacgccctcttaga                                                                                                                                                                                                                                                  >  1:374992‑M1/1‑266 (MQ=255)`  
                                                     `ttCTAATACGACTCACTAAAGGAGACACACCATGTTAAAACTGATTAAGAAGTTAGGCCAACTGCTGGTTCGTATGTACAACGTGGAAGCCAAGCGACTGAACGATGAGGCTCGTAAAGAGGCCACACAGTCACGCGCTCTGGCGATTCGCTCCAACGAACTGGCTGACAGTGCATCCACTAAAGTTACCGAGGCTGCCCGTGTGGCAAACCAAGCTCAACAGCTTTCCAAATTCTTTGAGTAATCAAACAGGAGAAACCATTATGatcgtttctgacatcgaagataacgccctcttaga                                                                                                                                                                                                                                                  >  1:457946‑M1/1‑266 (MQ=255)`  
                                                      `tCTAATACGACTCACTAAAGGAGACAGACCATGTTCAAACTGATTAAGAAGTTAGGCCAACTGCTGTTTCGTATGTACAACGTGGAAGCCAAGCGACTGAACGATGAGGCTCGTAAAGAGGCCACACAGTCACGCGCTCTGGCGATTCTCTCCAACGAACTGGCTGACAGTGCATCCACTAAAGTTACCGATGCTGCCCGTGTGGCAAACCAAGCTCAACAGCTTTCCAAATTCTTTGAGTAATCAAACAGGAGAAACCATTATGatcgtttctgacatcgaagctaacgccctcttagag                                                                                                                                                                                                                                                 <  1:213061‑M1/301‑37 (MQ=255)`  
                                                      `tCTAATACGACTCACTAAAGGAGACACACCATGTTCAAACTGATTAAGAAGTTAGGCCAACTGCTGGTTCGTATGTACAACGTGGAAGCCAAGCGACTGAACGATGAGGCTCGTAAAGAGGCCACACAGTCACGCGCTCTGGCGATTCGCTCCAACGAACTGGCTGACAGTGCATCCACTAAAGTTACCGAGGCTGCCCGTGTGGCAAACCAAGCTCAACAGCTTTCCAAATTCTTTGAGTAATCAAACAGGAGAAACCATTATGatcgtttctgacatcgaagctaacgccctcttagag                                                                                                                                                                                                                                                 <  1:462680‑M1/301‑37 (MQ=255)`  
                                                      `tCTAATACGACTCACTAAAGGAGACACACCATGTTCAAACTGATTAAGAAGTTAGGCCAACTGCTGGTTCGTATGGACAACGTGGAAGCCAAGCGGCTGAACGATGAGGCTCGTAAAGAGGACACACAGTCACGCGCTCTGGCGATTCGCTCCAACGAACTGGCTGACAGTGCATCCACTAAAGTTACCGAGGCTGCCCGTGTGGCAAACCAAGCTCAACAGCTTTCCAAATTCTTTGAGTAATCAAACAGGAGAAACCATTATGatcgtttctgacatcgaagctaacgccctcttagag                                                                                                                                                                                                                                                 <  1:109364‑M1/301‑37 (MQ=255)`  
                                                      `tCTAATACGACTCACTAAAGGAGACACACCAGTTTAAAACTGATTAAGAAGTTAGGCCAACTGCTGGTTCTTATGTACAACGTGGAAGCCAAGCGACTGAACGATGAGGCTCGTAAAGAGGCCACACAGTCACGCGCTCTGGCGATTCGCTGCAACGAACTGGCTGAAAGTGCATCCACTAAAGTTACCGAGGCTGCCCGTGTGGAAAACCAAGCTCAACAGCTTTCCAAATTCTTTGAGTAATCAAACAGGAGAAACCATTATGatcgtttctgacatcgaagctaacgccctcttagag                                                                                                                                                                                                                                                 <  1:125765‑M1/301‑37 (MQ=255)`  
                                                      `aataagagacagcactaaaggagacAAACCATGTTCAAACTGATTAAGAAGTTAGGCCAACTGCTGGTTCGTATGTACAACGTGGAAGCCAAGCGACTGAACGATGAGGCTCGTAAAGAGGCCACACAGTCACGCGCTCTGGCGATTCGCTCCAACGAACTGGCTGACAGTGCATCCACTAAAGTTACCGAGGCTGCCCGTGTGGCAAACCAAGCTCAACAGCTTTCCAAATTCTTTGAGTAATCAAACAGGAGAAACCATTATGatcgtttctgacatcgaagctaacgccctcttagag                                                                                                                                                                                                                                                 <  1:322800‑M1/289‑37 (MQ=255)`  
                                                        `tAATACGACTCACTATAGGAGACACACCATGTTCAAACTGATTAAGAAGTTAGGCCAACTGCTGGTTCGTATGTACAACGTGGAAGCCAAGCTACTGAACGATGAGGCTCGTAAAGAGGCCACACAGTCACGCGCTCTGGCGATTCGCTCCAACGAACTGGCTGACAGTGCATCCACTAAAGCTACCGAGGCTGCCCGTGTGGCAAACCAAGCTCAACAGCTTTCTAAAATCTTTGAGTAATCAAACAGGAGAAACCATTATGatcgtttctgatatagaagctaaagccctcttagagag                                                                                                                                                                                                                                               >  1:106304‑M1/1‑263 (MQ=255)`  
                                                                `cTCACTAAAGGAGACACACCATGTTCAAACTGATTAAGAAGTTAGGCAAACTGCTGGTTCGTATGTACAACGTGGAAGCCAAGCGACTGAACGATGAGGCTCGTAAAGAGGCCACACAGTCACGCGCTCTGGCGATTCGCTCCAACGAACTGGCTGACAGTGCATCCACTAAAGTTACCGAGGCTGCCCGTGTGGCAAACCAAGCTCAACAGCTTTCCAAATTCTTAGAGTAATCAAACAGGAGAAACCATTATGatcgtttctgacatcgaagctaacgccctcttagagagcgtcacta                                                                                                                                                                                                                                       <  1:475001‑M1/301‑47 (MQ=255)`  
                                                                  `cACTAAAGGAGACACCCCCTGTTCAAACTGATTAAGAAGTTAGGCCAACTGCTGGTTCGTATGTACAACGTGGAAGCCAAGCGACTGAACGATGAGGCTCGTAAAGAGGCCACACAGTCACGCGCTCTGGCGATTCGCTCCAACGAACTGGCTGACAGTGCATCCACTAAAGTTACCGAGGCTGCCCGTGTGGCAAACCAAGCTAACCAGCTTTCCAAATTCTTTGAGTAATCAAACAGGAGAAACCATTATGatcgtttctgacatcaaagctaacgaccacttagagctgtctcttatt                                                                                                                                                                                                                                     >  1:82429‑M1/1‑253 (MQ=255)`  
                                                                  `cACTAAAGGAGACACACCATGTTCAAACTGATTAAGAAGTTAGGCCAACTGCTGGTTCGTATGTACAACGTGGAAGCCAAGCGACTGAACGATGAGGCTCGTAAAGAGGCCACACAGTCACGCGCTCTGGCGATTCGCTCCAACGAACTGGCTGACAGTGCATCCACTAAAGTTACCGAGGCTGCACGTGTGGCAAACCAAGCTCAACAGCTTTCCAAATTCTTTGAGTAATCAAACAGGAGAAACCATTATGatcgtttctgacatcgaagctaacgccctcttagagagcgtcactaag                                                                                                                                                                                                                                     >  1:91492‑M1/1‑253 (MQ=255)`  
                                                                     `tAAAGGAGACACACCATGTTCAAACTGATTAAGAAGTTAGGCCAACTGCTGGTTCGTATGTACAACGTGGAAGCCAAGCGACTGAACGATGAGGCTCGTAAAGAGGCCACACAGTCACGCGCTCTGGCGATTCGCTCCAACGAACTGGCTGACAGTGCATCCACTAAAGTTACCGAGGCTGCCAGTGTGGCAAACCAAGCTCAACAGCTTTCCAAATTCTTTGAGTAATCAAACAGGAGAAACCATTATGatcgtttctgacatcgaagctaacgccctcttagagagcgtcactgagttc                                                                                                                                                                                                                                  <  1:197533‑M1/301‑52 (MQ=255)`  
                                                                      `aaaGGAGACACACCATGTTCAAACTGATTAAGAAGTTAGGCCAACTGCTGGTTCGGATGTACAACGTGGAAGCCAAGCGACTGAACGATGAGGCTCGTAAAGAGGCCACACAGTCACGCGCTCTGGCGATTCGCTCCAACGAACTGGCTTACAGTGCATCCACCAAAGTTACCGAGGCTGCCCGTGTGGCAAACCAAGCTCAACAGCTTTCCAAATTCTTTGAGTAATCAAACAGGAGAAACCATTATGatcgtttctgacatcgaagctaacgccctcttagagagcgtcactaagttcc                                                                                                                                                                                                                                 <  1:235915‑M1/301‑53 (MQ=255)`  
                                                                      `aaaGGAGACACAACATGTTCAAACGGAGTAAAAAGTTAGGCCAACTGCTGGTTCGTATTTACAACGTGGAAGCCAAGCGACTGAACGATGAGGCTCGTAAAGAGGCCACACAGTCACGCGCTCTGGCGATTCGCTCCAACGAACTGGCTGACAGTGCATCCACTACAGTTACCGAGGCTGCCCGTGTGGCAAACCAAGCTCAACAGCTTTCCAAACTCTTTGAGTAATCAAACAGTAGAAACCATTATGatcgtttctgacatcgaagctaacgccctcttatagagcgtcactaagttcc                                                                                                                                                                                                                                 <  1:394626‑M1/301‑53 (MQ=255)`  
                                                                        `aGGAGACACACCATGTTCAAACTGATTAAGAAGTTAGGCCAACTGCTGGTTCGTATGTACAACGTGGAAGCCAAGCGACTGAACGATGAGGCTCGTAAAGAGGCCACACAGTCACGAGCTCTGGCGATTCGCTCCAACGAACTGGCTGACAGTGCATCCACTAAAGTTACCGAGGCTGCCCGTGTGGCAAACCAAGCTCAACAGCTTTCCAAATTCTTTGAGTAATCAAACAGGAGAAACCATTATGatcgtttctgacatcgaagctaacgccctcttagagagcgtcactaagttccac                                                                                                                                                                                                                               >  1:339852‑M1/1‑247 (MQ=255)`  
                                                                         `tcggagatgtgtataagagacagattaagaagttaggccaactgcTGGTTCGTATGTACAACGTGGAAGCCAAGCGACTGAACGATGAGGCTCGTAAAGAGGCCACACAGTCACGCGCTCTGGCGATTCGCTCCAACGAACTGGCTGACAGTGCATCCACTAAAGTTACCGAGTCTGCACGTGT‑GCAAACCAAGCTCAACAGCTTTCCAAATTCTTTGAGTAATCAAACAGGAGAAATCAATATGatcgtttctgacatcgaagctaacgacctcttagagagcgtcactaagttccactg                                                                                                                                                                                                                             <  1:11327‑M1/279‑57 (MQ=255)`  
                                                                         `ggAGACACACCATGTTCAAACTGATTAAGAAGTTAGGCCAACTGCTGGTTCGTATGTACAACGTGGAAGCCACGCGACTGAACGATGAGGCTCGTAAAGAGGCCACACAGTCACGCGCTCTGGCGATTCGCTCCAACGAACTGGCTTACAGTGCATCCACTAAAGTTACCGAGGCTGCCCGTGTGTCCAACCAAGCTCAACAGCTTTCCAAATTCTTTGAGTAATCAAACAGGAGAAATCAATATGatcgtttctgacatcgaagctaacgccctcttagagagcgtcactaagttccact                                                                                                                                                                                                                              >  1:128863‑M1/1‑246 (MQ=255)`  
                                                                          `taagatacagatgttcaaactGATTAAGAAGTAAGGCCAACGGCTGTTTCGTATGTACAACGTGGAAGCCAAGCGACTGAACGATGAGGCTCGTAAAGAAGCCACACAGTCACGCGCTCTGGCGATTCGCTCCAACGAACTGGCTGAAAGTGCATCCACTAAAGTTACCGAGTCTGCCCGTGTGGCAAACCAAGCTCAACTGCTTTCCAAATTCTTTGAGTAATCAAACAGGAGAAACCATTATGatcgtttctgacatcgaagctaacgccctcttagagagcgtcactaagttccactg                                                                                                                                                                                                                             <  1:78840‑M1/291‑57 (MQ=255)`  
                                                                          `gagaCACACCATGTTCAAACTGATTAAGAAGTTAGGCCAACTGCTGGTTCGTATGTACAACGTGGAAGCCAAGCGACTGAACGATGAGGCTCGTAAAGAGGCCACACAGTCACGCGCTCTGGCGATTCGCTCCAACGAACTGGCTGACAGTGCATCCACTAAAGTTACCGAGGCTGCCCGTGTGGCAAACCAAGCTCAACAGCTTTCCAAATTCTTTTAGTAATCAAACAGGAGAAATCAATATGatcgtttctgacatcgaagataacgccctcttagagctgtctcttatacacatctc                                                                                                                                                                                                                             >  1:358027‑M1/1‑245 (MQ=255)`  
                                                                                 `acCATGTTCAAACTGATTAAGAAGTTAGGCCAACTGCTGGTTCGTATGTACAACGTGGAAGCCAAGCGACTGAACGATGAGGCTCGTAAAGAGGCCACACAGTCACGCGCTCTGGCGATTCGCTCCAACGAACTGGCTGACAGTGCATCCACTAAAGTTACCGAGGCTGCCCGTGTGGCAAACCAAGCTCAACAGCTTTCCAAATTCTTTGAGTACTCAAACAGGAGAAACCATTATGatcgtatctgacatcgaagttaacgccctcttagagagcgtcactaagttccactgctgtctc                                                                                                                                                                                                                      >  1:120962‑M1/1‑238 (MQ=255)`  
                                                                                     `ttttcaAACTGATTATGAAGTTAGGCCAACTGCTGGTTCGTATGTACAAAGTGGAAGCCAAGCTACTGAACGATGAGGCTCGTAAAGAGGCCACACAGTCACGCTCTCTGGCTATTCGCTCCAACGAACTGGCTGACAGTGCATCCACTAAAGTTACCGAGGCTGCCCGTGTTGCAAACCAAGCTCAACAGCTTTCCAAATTCTTTGAGTAATCAAACATGAGAAACCATTATGatcgtttctgacatcgaagctaacgccctcttagagagcgtcactaagttccactgcggggttatct                                                                                                                                                                                                                  <  1:86750‑M1/299‑68 (MQ=255)`  
                                                                                     `ttttcaAACTGATTAAGAAGTTATGCCAACTGCTGGTTCGTATGTACAACGTGGAAGCCAAGCGACTGAACGATGAGGCTCGTAAAGAGGCCACACAGTCACGCGCTCTGGCGATTCTCTCCAACGAACTGGCTGACAGTGCATCCACTAAAGTTACCGAGGCTGCCCGTGTGGCAAACCAAGCTCAACAGCTTTCCAAATTCTTTGAGTAATCAAACAGGAGAAATCAATATGatcgtttctgacatcgaagctaacgccctcttagagagcgtcactaagttccactgcggggttatct                                                                                                                                                                                                                  <  1:159106‑M1/299‑68 (MQ=255)`  
                                                                                     `tGTTCAAACTGATTAAGAAGTTAGGCCAACTGCTGGTTCGTATGTACAACGTGGAAGCCAAGCGTCTGGACGATGAGTCTCGTAAAGAGGCCACACAGTCACGCGCTCTGGCGATTCGCTCCAACGAACTGGCTGACAGTGCATCCACTAAAGTTACCGAGGCCGCCCGTGTGGCAAACCAAGCTCAACAGCTTTCCAAATTCTTTTAGTAATCAAACAGGAGAAACCATTATGatcgtttctgacatcgaagctaacgccctcttagagagcgtcactaagttccactgcggggttatct                                                                                                                                                                                                                  <  1:227685‑M1/301‑68 (MQ=255)`  
                                                                                     `tGTTCAAACTGATTAAGAAGTTAGGCCAACTGCTGGTTCGTATGTACAACGTGGAAGCCAAGCGACTGAACGATGAGGCTCGTAAAGAGGCCACACAGTCACGCGCTCTGGCGATTCGCTCCAACGAACTGGCTGACAGTGCATCCACTAAAGTTACCGAGGCTGCCCGTGTGGCAAACCAAGCTCAACAGCTTTCCAAATTCTTTGAGTAATCAAACAGGAGAAACCATTATGatcgtttctgacatcgaagctaacgccctcttagagagcgtcactaagttccactgcggggttatct                                                                                                                                                                                                                  <  1:182180‑M1/301‑68 (MQ=255)`  
                                                                                       `ttttataagggacagaagttaggccaactgcTGGTTCGTATGTACAACGTGGAAGCCAAGCGACTTAACGATGAGGCTCGTAAAGAGGCCACACAGTCACGCGCTCTGGCGATTCGCTCCAACGAACTGGCTGACAGTGCATCCACTAAAGTTACCGAGGCTGCCCGTGTGGCAAACCAAGCTCAACAGCTTTCCAAATTCTTTGAGTAATCAAACAGGAGAAACCATTATGatcgtttctgacatcgaagctaacgccctcttagagagcgtcactaagttccactgcggggttatctac                                                                                                                                                                                                                <  1:32885‑M1/288‑70 (MQ=255)`  
                                                                                          `agacaggttaagaagtTAGGCCAACTGCTGGTTCGTATGTACAACGTGGAAGCCAAGCGACTGAACGATGAGGCTCGTAAAGAGGCCACACAGGCACGCGCTCTGGCTATTCGCTCCAACGAACTGGCTGACAGTGCATACACTAAAGTTACCGAGGCTGCCCGTGTGGCAAACCAAGCTCAACAGCTTTCCAAATTCTTTGAGTAATCAAACACGAGAAACCAGTATGatcgtttctgacatcgaagctaacgccctcttagagagcgtcactaagttccactgcggggttatctacgac                                                                                                                                                                                                             <  1:245824‑M1/294‑73 (MQ=255)`  
                                                                                             `cTGATTAAGAAGTTAGGCCAACTGCTGGTTCGTATGTACAACGTGGAAGCCAAGCGCCTGAACGATGAGGCTCGTAAAGAGGCCACACAGTCACGCGCTCTGGCGATTCGCTCCAACGAACTTGCTGACAGTGCATCCACTAAAGTTTCCGAGGCTGCCCGTCTGCCACTCCATCCTCAACAGCTTTCCAAATTCTTTGAGTAATCAAACCGGTGCACCCATTTTGatcgtttctgacatcgacgctaccgccctcttagagggcgtcactacgttcccctccgggtttactgtctcttat                                                                                                                                                                                                          >  1:240841‑M1/1‑226 (MQ=255)`  
                                                                                               `gATTAAGAAGTTAGGCCAAATGCTGGTTCGTATGTACAACACGGAAGCCAAGCGACTGGACGATGAGACGCGTAAAGAGGCCACACAGTCACGCGCTCTGGCGATTCGCTCCAACGAACTGGCTGAAAGTGGATCCACTAAAGTTACCGAGGCTGGCCGTGTGGCAAACAAAGTTAATCAGCGTTCCAAATTCTTTGAGTAATAAAACAGGAGAAATCAATATGatcgtttctgacatcgaagctaacgccctcttagagagcgtcactaagttccactgcggggttatctacgactactc                                                                                                                                                                                                        <  1:333888‑M1/301‑78 (MQ=255)`  
                                                                                               `aaataagAAGTTAGGCCAACTGCTGGTTCGTATGTACAACGTTGAAGCCAAGCGACTGAACGATGAAGCTCGTAAAGAGGCCACGCAGTCACGCGCTCTGGCGATTCGCTCCAACGAACTGGCTGACAGTGCATCCACTAAAGTTACCGAGGCTGCCCGTGTGGCAAACCAAGCTCAACAGCTTTCCAAATTCTTTGAGTAATCAAACAGGAGAAACCATTATGatcgtttctgacatcgaagctaacgccctcttagagagcgtcactaagttccactgcggggttatctacgactactc                                                                                                                                                                                                        <  1:454607‑M1/298‑78 (MQ=255)`  
                                                                                                  `aaagaagtTAGGCCAACTGCTGGTTCGTATGTACAACGTGGAAGCCAAGCGACTGAACGATGAGGCTCGTAAAGAGGCCACACAGTCACGCGCTCTGGCGATTCGCTCCAACGAACTGGCTGACAGTGCATCCACTAAAGTTACCGAGGCTGCCCGTGTGGCAAACCAAGCTCAACAGCTTTCCAAATTCTTTGAGTAATCAAACAGGAGAAACCATTATGatcgtttctgacatcgaagctaacgcactcttagagagcgtcactaagttccactgcggggttatctacgactactccac                                                                                                                                                                                                     <  1:481250‑M1/300‑81 (MQ=255)`  
                                                                                                     `gggctcggagatgtgtataagagacaggtacaacgtggaagccaagcgactgaacGATGAGGCTCGTAAAGAGGCCACACAGTCACGCGCTCTGGCGATTCGCTCCAACGAACTGGCTGACAGTGCATCCACTAAAGTTACCGAGGCTGCCCGTGTGGCAAACCAAGCTCAACAGCTTTCCAAATTCTTTGAGTAATCAAACAGGAGAAACCATTATGatcgtttctgacatcgaagctaacgccctcttagagagcgtcactaagttccactgcggggttatctacgactactccaccgc                                                                                                                                                                                                  <  1:22288‑M1/274‑84 (MQ=255)`  
                                                                                                     `gaagTTAGGCCAACTGCTGGTTCGTATGTACAACGTGGAAGCCAAGCGACTGAACGATGAGGCTCGTAAAGAGGCCACACAGTCACGCGCTCTGGCGATTCGCTCCAACGAACTGGCTGACAGTGCATCCACTAAAGTTACCGAGGCTGCCCGTGTGGCAAACCAAGCTCAACAGCTTTCCAAATTCTTTGAGTAATCAAACAGGAGAAACCATTATGatcgtttctgacatcgaagctaacgccctcttagagagcgtcactaagttccactgcggggttatctacgactactccaccgc                                                                                                                                                                                                  >  1:116434‑M1/1‑218 (MQ=255)`  
                                                                                                      `aagTTAGGCCAACTGCTGGTTCGTATGTACAACGTGGAAGCCAAGCGACTGAACGATGAGGCTCGTAAAGAGGCCACACAGTCACGCGCTCTGGCGATTCGCTCCAACGAACTGGCTGACAGTGCATCCACTAAAGTTACCGAGGCTGCCCGTGTGGCAAACCAAGCTCAACAGCTTTCCAAATTCTTTGAGTAATCAAACAGGAGACACCATTATGatcgtttctgacatcgaagctaacgccctcttagagagcgtcactaagttccactgcggggttatctctgtctcttatccccat                                                                                                                                                                                                 >  1:361915‑M1/1‑217 (MQ=255)`  
                                                                                                         `taaggCCAACTTCTGGTTCGTATGTACAACGTGGAAGCCAAGCGAATGAACGAGGAGGTGCGTAAAGAGGCCACACAGTCACGCGCTCTGGCGATTCGCTCCAACGAACTGGCTGACAGTGCATCCAATAAAGTTACCGAGGCTGACCGTGTGGCAAAACAAGCTCAACAGCATTCCAAATAATATGAGTAATC‑AATAGGAGAAATCAATATGatcgtttctgacatagaagctaacgcccgcttagagagcgtcactaagttccactgcggggttatctacgactactccaccactgagt                                                                                                                                                                                             <  1:254287‑M1/299‑89 (MQ=255)`  
                                                                                                            `ggCCAACTGCTGGTTCGTATGTACAACGTGGAAGCCAAGCGACTGAACGATGAGGCTCGTAAAGAGGCCACACAGTCACGCGCTCTGGCGATTCGCTCCAACGAACTGGCTGACAGTGCATCCACTAAAGTTACCGAGGCTGCCCGTGTGGAAAACCAAGCTCAACAGCTTTCCAAATTCTTTGAGTAATCAAACAGGAGAAACCATTATGatcgtttctgacatcgaagctaacgccctcttagagagcgtcactaagttccactgcggggttatctacgactactccaccgctgagtac                                                                                                                                                                                           <  1:378599‑M1/301‑91 (MQ=255)`  
                                                                                                               `tgtataagagacagtatgtacaacgtgGAAGCCAAGCGACTGAACGATGAGGCTCGTAAAGAGGCCACACAGTCACGCGCTCTGGCGATTCGCTCCAACGAACTGGCTGACAGTGCATCCACTAAAGTTACCGAGGCTGCCCGTGTGGCAAACCAAGCTCAACAGCTTTCCAAATTCTTTGAGTAATCAAACAGGAGAAACCATTATGatcgtttctgacatcgaagctaacgccctcttagagagcgtcactaagttccactgcggggttatctacgactactccaccgctgagtacgta                                                                                                                                                                                        <  1:250035‑M1/288‑94 (MQ=255)`  
                                                                                                               `cAACTGCTGGTTCGTATGTACAACGTGGAAGCCAAGCGACTGAACGATGAGGCTCGTAAAGAGGCCACACAGTCACGCGCTCTGGCGATTCGCTCCAACGAACTGGCTGACAGTGCATCCACTAAAGTTACCGAGGCTGCCCGTGTGGCAAACCAAGCTCAACAGCTTTCCAAATTCTTTGAGTAATCAAACAGGAGAAATCAATATGatcgtttctgacatcgaagctaacgccctcttagagagcgtcactaagttccactgcagggttatctacgactactccaccgctgagtacgta                                                                                                                                                                                        <  1:276333‑M1/301‑94 (MQ=255)`  
                                                                                                                     `ctgGTTCGTATGTACAACGTGGAAGCCAAGCGACTGAACGATGAGGCTCGTAAAGAGGCCACACAGTCACGCGCTCTGGCGATTCGCTCCAACGAACTGGCTGACAGTGCATCCACTAAAGTTACCGAGGCTGCCCGTGTGGCAAACCAAGCTCAACAGCTTTCCAAATTCTTTGAGTAATCAAACAGGAGAAACCATTATGatcgtttctgacatctaagctaacgccctcttagagagcgtcactaagttccactgcggggttatctacgactactccaccgctgagtacgtaagctac                                                                                                                                                                                  <  1:402100‑M1/301‑100 (MQ=255)`  
                                                                                                                     `ctgGTTCGTATATACAACGTGGAAGCCAAGCGACTGAACGAGGAGGCTCGTAAAGAGGCCACACAGTCACGCGCTCTGGCGATTCGCTCCAACGAACTGGCTGACAGTGCATCCACTAAAGTTACCGAGGCTGCACGTGTGGCAAACCAAGCTCAACAGCTTTCCAAATTCTTTGAGTAATCAAACAGGAGAAACCATTATGatcgtttctgacatcgatgctaacgccctcttagagagcgtcactaagttccactgcggggttagctacgactactccacctctgagtacgtaagctac                                                                                                                                                                                  <  1:165090‑M1/301‑100 (MQ=255)`  
                                                                                                                         `ttCGTATGTACAACGTGGAAGCCAAGCGACTGAACGATGAGGCTCGTAAAGAGGCCACACAGTCACGAGCTCTGGCGATTCGCTCCAACGAACTGGCTGACAGTGCATCCACTAAAGTTACCGAGGCTGCCCGTGTGGCAAACCAAGCTCAACAGCTTTCCAAATTCTTTGAGTAATCAAACAGGAGAAATAAATATGatcgtttatgacatcgaagctaacgccctcttagagagcgtcactaagttccactgcggggttatctacgactaatccaccgctgcgtccctgtctcttatac                                                                                                                                                                              >  1:367126‑M1/1‑198 (MQ=255)`  
                                                                                                                            `gTATGTACAACGTGGAAGCCAAGCGACTGAACGATGAGGCTCGTAAAGAGGCCACACAGTCACGCGCTCTGGCGATTCGCTCCAACGAACTGGCTGACAGTGCATCCACTAAGGTTACCGAGGCTGCCCGTGTGGCAAACCAAGCTCAACAGCTTTCCAAATTCTTTGAGTAATCAAACAGGAGAAACCATTATGatcgtttctgacatcgaagctaacgccctcttggagagcgtcactaagttccactgcggggttatctacgactactccaccgctgagtacgtaagctaccgtccga                                                                                                                                                                           >  1:429330‑M1/1‑195 (MQ=255)`  
                                                                                                                            `gTATGTACAACGTGGAAGCCAAGCGACTGAACGATGAGGCTCGTAAAGAGGCCACACAGTCACGCGCTCTGGCGATTCGCTCCAACGAACTGGCTGACAGTGCATCCACTAAAGTTACCGAGGCTGCCCGTGTGGCAAACCAAGCTCAACAGCTTTCCAAATTCTTTGCGTAATCAAACAGGAGAAATCAATATGatcgtttctgacatcgaagctaacgccctcttagagagcgtcactaagttccactgcggggttatctacgactactccaccgctgagtacgtaagctaccgtccga                                                                                                                                                                           >  1:155680‑M1/1‑195 (MQ=255)`  
                                                                                                                               `tGTACAACGTGGAAGCCAAGCGACTGAACGATGATGCTCGTAAAGAGGCCACACAGTCACGCGCTCTGGCGATTCGCTCCAACTAAATGGCTGACAGTGCATCCACTAAAGTTACCGAGGCTGCGCGTGTGGCAAACCAAGCTCAACAGCTTTCCAAATTCTTTGAGTAATCAAACAGGAGAAACCATTATGatcgtttcttacatcgaagctaacgccctcttagatagcttcactaagttccactgcggggttatctacgactactccaccgctgagtacgtaagctaccgtccgagtg                                                                                                                                                                        <  1:510045‑M1/301‑110 (MQ=255)`  
                                                                                                                               `tGTACAACGTGGAAGCCAAGCGACTGAACGATGAGGCTCGTAAAGATGCCACACAGTCACGCGCTCTGGCTATTCGCTCCAACGAACTGGCTGACAGTGCATCCACTAAAGTTACCGAGGCTGCCCGTGTGGCAAACCAAGCTCAACAGCTTTCCAAATTCTTTGAGTAATCAAACAGGAGAAATCAATATGatcgtttctgacatcgaagctaacgccctcttagagagcgtcactaagttccactgcggggttatctacgactactccaccgctgagtacgtaagctaccgtccgagtg                                                                                                                                                                        <  1:465259‑M1/301‑110 (MQ=255)`  
                                                                                                                                 `gaaaacgtGGAAGCCAAGCGACTGAACGATGAGGCTCGTAAAGAGGCCACACAGTCACGCGCTCTGGCGATTCGCTCCAACGAACTGGCTGACAGTGCATCCACTAAAGTTACCGAGGCTGCCCGTGTGGCAAACCAAGCTCAACAGCTTTCCAAATTCTTTGAGTAATCAAACAGGAGAAACCATTATGatcgtttctgacatcgaagctaacgccctcttagagagcgtcactaagttccactgcggggttatctacgactactccaccgctgagtacgtaagctaccgtccgagtgac                                                                                                                                                                      <  1:470026‑M1/298‑112 (MQ=255)`  
                                                                                                                                 `aacAACGTGGAATCCAAGCGACTGAACGATGAGGCTCGTAAAGAGGCCACACAGTCACACGCTCTGGCGATTCGCTCCAACGAACTGGCTGACAGTGCATCCACTAAAGTTACCGAGGCTGCCCGTGTGGCAAACCAAGCTCAACAGCTTTCCAAATTCTTTGAGTAATCAAACAGGAGAAACCATTATGatcgtttctgacatcgaagctaacgccctcttagagagcgtcactaagttccactgcggggttatctacgactactccaccgctgagtacgtaagctaccgtccgagggac                                                                                                                                                                      <  1:383380‑M1/300‑112 (MQ=255)`  
                                                                                                                                     `aCGTGGAAGCCAAGCGACTGAACGATGAGGCTCGTAAAGAGGCCACACAGTCACGCGCTCTGGCGATTCGCTCAAACGAACTGGCTGACAGTGCATCCACTAAAGTTACCGAGGCTGCCCGTGTGGCAAACCAAGCTCAACAGCTTTCCAAATTCTTTGAGTAATCAAACAGGAGAAACCATTATGatcgtttctgacatagaagctaacgccctcttagagagcgtcactaagttccactgcggggttatctacgactactccaccgctgagtacgtaagctaccgtccgagtgacttcg                                                                                                                                                                  >  1:298398‑M1/1‑186 (MQ=255)`  
                                                                                                                                      `cGTGGAAGCCAAGAGACTGAACGATGAGGCTCGTAAAGAGGCCACACAGTCACGCGCTCGGGCGATTCGCGCCAACGAACTGGCTGACAGTGCATCCACTAAAGTTACCGAGGCTGCCCGTGTGGCAAACCAAGCTCAACAGCTTTCCAAATTCTTTGAGTAATCAAACAGGAGAAACCATTATGatcgtttctgacatcgaagctaacgccctcttagagagcgtaactaagttccactgcggggttatctacgactactccaccgctgagtacgtaagctaccgtccgagtgacttcgg                                                                                                                                                                 <  1:349124‑M1/301‑117 (MQ=255)`  
                                                                                                                                          `gAAGCCAAGCGACTGAACGATGAGGCTCGTAAATAGGCCACACAGTCACGCGCTCTGGCGATTCGATCCAACGAACTGGCTGACAGTGCATCCACTAAAGTTACCGAGGCTGCCCGTGTGGCGAACCAAGCTCAACAGCTTTCCAAATTCTTTGAGTAATCAAACAGGAGAAACCATTATGatcgtttctgacatcgaagctaacgccctcttatagagcgtcactaagttccactgcggggttatctacgactactccaccgctgagtacgtaagctaccgtccgagtgacttcggtgcg                                                                                                                                                             <  1:270803‑M1/301‑121 (MQ=255)`  
                                                                                                                                              `ccAAGCGACTGAACGATGAGGCTCGTAAAGAGGCCACACAGTCACGAGCTCTGGCGATTCGCTCCAACGAACTGGCTGACAGTGCATCCACTAAAGTTAACGAGGCTGCCCGTGTGGCAAACCAAGCTCAACAGCTTTCCAAATTCTTTGAGTAATCAAACAGGAGAAACCATTATGatcgtttctgacatcgaagctaacgccctcttagagagcgtcactaagttccactgcggggttatctacgactactccaccgctgagtacgtaagctaccgtccgagtgacttcggtgcgtatc                                                                                                                                                         <  1:369583‑M1/301‑125 (MQ=255)`  
                                                                                                                                              `ccAAGCGACGGAACGATGAGGCTCGTAAAGAGGCCACACAGTCACGCGCTCTGGCGATTCGCTCCAACGAAATGGCTGACAGTGCATCCACTAAAGTTACCGAGGCTGCCCGTGTGGCAAACCAAGCTCAACAGCTTTCCAAATTCTTTGAGTAATCAAACAGGAGAAACCATTATGatcgtttctgacatcgaagctaacgccctcttagagagcgtcactaagttccactgcggggttatctacgactactccaccgctgagtacgtaagctaccgtccgagtgacttcggtgcgtatc                                                                                                                                                         <  1:279757‑M1/301‑125 (MQ=255)`  
                                                                                                                                                  `gCGACTGAACGATGAGGCTCGTAAAGAGGCCACACAGTCACGCGCTCTGGCGATTCGCTCCAACGAACTGGCTGACAGTGCATCCACTAAAGTTACCGAGGCTGCCCGTGTGGCAAACCAAGCTCAACAGCTTTCCAAATTCTTTGAGTAATCAAACAGGAGAAATCAATATGatcgtttctgacatcgaagctaacgccctcttagagagcgtcactaagttccactgcggggttatctacgactactccaccgctgagtacgtaagctaccgtccgagtgacttcggtgcgtatctgga                                                                                                                                                     <  1:441916‑M1/301‑129 (MQ=255)`  
                                                                                                                                                               `aagGCTCCAAAAGAGTCCACATAGTAACGCGGTCAGGCGATTCGCTCCAACAAACTGGCTGACAGTGCATCCACTAAAGTTACCGAGGCTGCCCGTGTGTCAAACAAAGCTAAACAGCTTTCCAAATTCGATGAGTAATCAAACAGGAGAAATCAATATGatcgtttctgacatcgaagctaacgccctcttagagagcgtcactaagttccactgcggggtgatctacgactactccaccgctgagtacgtaagctaccgtccgagtgacttcggtgcgtatctggatgcgctggaagcc                                                                                                                                        <  1:353444‑M1/300‑142 (MQ=255)`  
                                                                                                                                                                  `gCTCGTAAAGAGGCCACACAGTCACGCGCTCTGGCGATTCGCTCAAACGAACTGGCTGACAGAGCATCCACTAAAGATACCGAGTCTGCCCGATTGGCAAACCATGCTCAACAGCTTTCCAAATTCTTAGAGTAATCAAACAGGAAAAACCATTATGgtcgcttatgtaatcgaagctaaagccctcttagatgtcgtcactcagttcaactgcggggttatctacgactactccatcgctgagtacgtaagctaccgtccgagtgacttcggtgcgaatatggatgctgtcacttataca                                                                                                                                     >  1:45035‑M1/1‑157 (MQ=255)`  
                                                                                                                                                                           `gagGCCACACAGTCACGCGCTCTGGCGATTCGCTCCAACGAACTGGCTGACAGTGCATCCACTAAAGTTACCGAGGCTGCCCGTGTGGCAAACCAAGCTCTACAGCTTTCCAAATTCTTTGAGTAATCAAACAGGAGAAACCATTATGatcgtttctgacatcgaagctaacgccctcttaacgagcgtccctaagttccactgcggggttatctacgactactccaccgctgagtccgtaagctaccgtccgagtgacttcggtgcgtatctggatgcgctggaatccgaggttgcacga                                                                                                                            >  1:28898‑M1/1‑148 (MQ=255)`  
                                                                                                                                                                                  `aacagTGACGCGGTCTGGCGATTGGCTCCAACGAACTGGCTGACAGTGCATCCACTAAAGTTACCGAGGCTGCACGTGTGGCAAACCAATCTCAACAGCTTTCCAAATTCTTTGAGTAATCAAACAGGAGAAACCATTATGatcgtttctgacatcgaagctaacgccctcttagagagcgtcactaagttccactgcggggttatctacgactactccaccgctgagtacgtaagctaccgtccgagtgacttcggtgcgtatctggatgcgctggaagccgaggttgcacgaggcggtc                                                                                                                     <  1:495725‑M1/300‑161 (MQ=255)`  
                                                                                                                                                                                    `caGTCACGCGCTCTGGCGATTCGCTCCAACGAACTGGCTGACAGTGCATCCACTAAAGTTACCGAGGCTGCCCGTGTGGCAAACCAAGCTCAACAGCTTTCCAAATTCTTTGAGTAATCAAACAGGAGAAACCATTATGatcgtttctgacatcgaagctaacgccctcttagagagcgtcactaagttccactgcggggttatctacgactactccaccgctgagtacgtaagctaccgtccgagtgacttcggtgcgtatctggatgcgctggaagccgaggttgcacgaggcggtctt                                                                                                                   <  1:142358‑M1/301‑163 (MQ=255)`  
                                                                                                                                                                                      `gTCACGCGCTCTGGCGATTCGCTCCAACGAACTGGCTGACAGTGCATCAACTAAAGTTACCGAGGCTGCCCGTGTGGCAAACCAAGCTCAACAGCTTTCCAAATTCTTTGAGTAATCAAACAGGAGATACCATTATGatcgtttctgacatcgaagctaacgccctcttagagagcgtcactaagttccactgcggggttatctccgactactccaccgctgagtacgtaagctaacgtcagagtgaattcggtgcgtatctggatgcgctggaagccgaggttgcacgaggcggtcttat                                                                                                                 >  1:68192‑M1/1‑137 (MQ=255)`  
                                                                                                                                                                                        `cACGCGCTCGGGCGATTCGCTCCAACGAACTGGCTGACAGTGCATCCACTAAAGTTACCGAGGCTGCCCGTGTGGCAAACCAAGCTCAACAGCTTTCCAAATTCTTTGAGTAATCAAACAGGAGAAACCATTATGatcgtttctgacatcgaagctaacgccctcttagagagcgtcactaagttccactgcggggttatctactactactccaccgctgagtacgtaagctaccgtccgagtgacttcggtgcgtatctggatgcgctggaagccgaggttgcacgaggcggtcttattg                                                                                                               <  1:54065‑M1/301‑167 (MQ=255)`  
                                                                                                                                                                                              `ctctGGCGATTCGCTCCAACGAACTGGCTGACAGTGCATCCACTAAAGTTACCGAGGCTGCCCGTGTGGCAAACCAAGCTCAACAGCTTTCCAAATTCTTTGAGTAATCAAACAGGAGAAACCATTATGatcgtttctgacatcgaagctaacgccctcttagagagcgtcactaagttccactgcggggttatctacgactactccaccgctgagtacgtaagctaccgtccgagtgacttcggtgcgtatctggatgcgctggaagccgaggttgcacgaggcggtcttattgtgttcc                                                                                                         >  1:438063‑M1/1‑129 (MQ=255)`  
                                                                                                                                                                                                `ctGGCGATTCGCTCCACCGAACTGGCTGACAGTGCATCCACTAAGGTTACCGAGGCTGCCCGTGTGGCAAACCAAGCTCAACAGCTTTCCAAATTCTTTGAGTAATCAAACAGGAGAAACCATTATGatcgtttctgacatcgaagctaacgccctcttagagagcgtcactaagttccactgcggggttatctacgactactccaccgctgagtacgtaagctaccgtccgagtgacttcggtgcgtatctggatgcgctggaagccgaggttgcacgaggcggtcttattgtgttccac                                                                                                       <  1:267374‑M1/301‑175 (MQ=255)`  
                                                                                                                                                                                                `ctGGCGATTCGCTCCAACGAACTGGCTGACAGTGCATCCACTAAAGTTACCGAGGCTGCCCGTGTGGCAAACCAAGCTCAACAGCTTTCCAAATTCTTTGAGTAATCAAACAGGAGAAACCATTATGatcgtttctgacatcgaagctaacgccctcttagagagcgtcactaagttccactgcggggttatctacgactactccaccgctgagtacgtaagctaccgtccgagtgacttcggtgcgtatctggatgcgctggaagccgaggttgcacgaggcggtcttatttctgtctct                                                                                                       >  1:172205‑M1/1‑127 (MQ=255)`  
                                                                                                                                                                                                                `aCGAACTGGCTGACAGTGCATCCACTAAAGTTACCGAGGCTGCCCGTGTGGCAAACCAAGCTCAACAGCTTTCCAAATTCTTTGAGTAATCAAACAGGAGAAACCATTATGatcgtttctgacatcgaagctaacgccctcttagagagcgtcactaagttccactgcggggttatctacgactactccaccgctgagtacgtaagctaccgtccgagtgacttcggtgcgtatctggatgcgctggaagccgaggttgcacgaggcggtcttattatgttccacaacggtcacaagtatg                                                                                       >  1:146138‑M1/1‑111 (MQ=255)`  
                                                                                                                                                                                                                 `cGAACTGGCTGACAGTGCATCCACTAAAGTTACCGAGGCTGCCCGTGTGGCAAACCAAGCTCAACAGCTTTCCAAATTCTTTGAGTAATCAAACAGGAGAAATCAATATGatcgtttctgacatcgaagctaacgccctcttagagagcgtcactaagttccactgcggggttatctacgactactccaccgctgagtacgtaagctaccgtccgagtgacttcggtgcgtatctggatgcgctggaagccgaggttgcacgaggcggtcttattgtgttccacaacggtcacaagtatga                                                                                      <  1:74277‑M1/301‑192 (MQ=255)`  
                                                                                                                                                                                                                   `tataagagacaggtgcatccactaaAGTGACCGAGGCTGCCCGTGTGGCAAACCAAGCTCAACAGCTTTCCAAATTCTTTGAGTAATCAAACAGGAGAAACCATTATGatcgtttctgacatcgaagctaacgccctcttagagagcgtcactaagttccactgcggggttatctacgactactccaccgctgagtacgtaagctaccgtccgagtgacttcggtgcgtatctggatgcgctggaagccgaggttgcacgaggcggtcttattgtgttccacaacggtcacaagtatgacg                                                                                    <  1:151022‑M1/289‑194 (MQ=255)`  
                                                                                                                                                                                                                     `cTGGCTGACAGTGCATCCACTAAAGTTACCGAGGCTGCCCGTGTGGCAAACCAAGCTCAACAGCTTTCCAAATTCTTTGAGTAATCAAACAGGAGAAACCATTATGatcgtttctgacatcgaagctaacgccctcttagagagcgtcactaagttccactgcggtgttatctacgactactccaccgctgagtacgtaagctaccgtccgagtgacttcggtgcgtatctggatgcgctggaagccgaggttgcacgaagcggtcttattgtgttccacaacggtcacaagtatgacgtt                                                                                  >  1:58415‑M1/1‑106 (MQ=255)`  
                                                                                                                                                                                                                     `cTGGCTGACAGTGCATACACTAAAGTTACCGAGGCTGCCCGTGTGGCAAACCAAGCTCAACAGCTTTCCAAATTCTTTGAGTAATCAAACAGGAGAAATCAATATGatcgtttctgacatcgaagctaacgccctcttagagagcgtcactaagttccactgcggggttatctacgactactccaccgctgagtacgtaagctaccgtccgagtgacttcggtgcgtatctggatgcgctggaagccgaggttgcacgaggcggtcttattgtgttccacaacggtcacaagtatgaaggt                                                                                  >  1:41569‑M1/1‑106 (MQ=255)`  
                                                                                                                                                                                                                        `gCTGACAGTGCATCCACTAAAGTTACCGAGGCTGCCCGTGTGGCAAACCAAGCTCAACAGCTTTCCAAATTCTTTGAGTAATCAAACAGGAGAAACCATTATGatcgtttctgacatcgaagctaacgccctattagagagcgtcactaagttccactgcggggttatctacgactactccaccgctgagtacgtaagctaccgtccgagtgacttcggtgcgtatctggatgcgctggaagccgaggttgcacgaggcggtcttattgtgttacaaaactgtcacaagtatgacgttcct                                                                               >  1:283995‑M1/1‑103 (MQ=255)`  
                                                                                                                                                                                                                             `cAGTGCATCCACTAAAGTTACCGAGGCTGCCCGTGTGACAAACCAAGCTCAACAGCTTTCCAAATTCTTTGAGTAATCAAACAGGAGAAACCATTATGatcgtttctgacatcgcagctaacgccctcttagagagcggcactaagttccactgcggggttatctacgactactccaccgctgagtacgtaagctaccgtccgagtgacttcggtgcgtatctggatgcgctggaagccgcggttgcacgaggccgtcttattgttttccccaacggtcgcaagtatgacgttccttcatt                                                                          >  1:101400‑M1/1‑98 (MQ=255)`  
                                                                                                                                                                                                                               `gTGCATCCACTAAAGTTACCGAGGCTGCCCGTGTGGCAAACCAAGCTCAACAGCTTTCCAAATTCTTTGAGTAATCAAACAGGAGAAACCATTATGatcgtttctgacatcgaagctaacgccctcttagagagcgtcactaagttccactgcggggttatctacgactactccaccgctgagtacgtaagctaccgtccgagtgacttcggtgcgtatctggatgcgctggaagccgaggttgcacgaggcggtcttattgtgttccacaacggtcacaagtatgacgttcctgcattga                                                                        >  1:232597‑M1/1‑96 (MQ=255)`  
                                                                                                                                                                                                                                 `gCATCTACTAAAGTTACCGAGGCTGCCCGTGTGGCAAACCAAGCTCAACAGCTTTCCAAATTCTTTGAGTAATCAAACAGGAGAAATCAATATGatcgtttctgacatcgaagctaactccctcttagagagcgtcactaagttccactgcggggttatctacgactactccaacgctgagtacgtaagctaccgtccgagtgacttcggtgcgtatctggatgcgcttgaagccgaggttgcacgaggcggtcttattgtgttccacaactctcacaagtatgacgttcctgcattgacc                                                                      >  1:501458‑M1/1‑94 (MQ=255)`  
                                                                                                                                                                                                                                 `ccaTCCACTAAAGTTACCGGGGCTGCCCGTGTGGCAAACCAAGCTCAACAGCTTTCCAAATTCTTTGATTAATCAAACAGGAGAAACCATTATGatcgtttctgacatcgaagctaacgccctcttagagagcgtcactaagttccactgcggggttatctacgactactccaccgctgagtacgtaagctaccgtccgagtgacttcggtgcgtatctggatgcgctggaagccgaggttgcacgaggcggtcttattgtgttccacaacggtcacaagtatgacgttcctgcattgacc                                                                      <  1:192819‑M1/300‑208 (MQ=255)`  
                                                                                                                                                                                                                                         `aaaagTTACCGAGGCTGCCCGTGTGGCAAACCAAGCTCAACAGCTTTCCAAATTCTTTGAGTAATCAAACATGAGAAATAAATATGatagttgctgaaatcgaagctaacgccctcttagagagcttcacgaagttccactgcggggttatctacgactactccaccgctgagtacgtaagctaccgtcctagtgacttcggtgcgtatctggatgcgctggaagccgaggttgcacgaggcggtcttattgtgtttcacaacggtcacaagtatgacgttcctgcattgaccaaactggc                                                              <  1:505372‑M1/300‑216 (MQ=255)`  
                                                                                                                                                                                                                                               `tACCGAGGCTGCCCGTGTGGCAAACCAAGCTCAACAGCTTTCCAAATTCTTTGAGTAATCAAACAGGAGAAATCAATATGatcgtttctgacatcgaagctaacgccctcttagagagcgtcactaagttccactgcggggttatctacgactactccaccgctgagtacgtaagctaccgtccgagtgacttcggtgcgtatctggatgcgatggaagccgaggttgcacgaggcggtcttattgtgttccacaacggtcacaagtatgacgttcctgcattgaccaaactggcaaagtt                                                        >  1:187709‑M1/1‑80 (MQ=255)`  
                                                                                                                                                                                                                                               `tACCGAGGCTGCCCGTGTGGCAAACCAAGCTCAACAGCTTTCCAAATTCTTTGAGTAATCAAACAGGAGAAACCATTATGatcgtttctgacatcgaagctaacgccctcttagagagcgtcactaagttccactgcggggttatctacgactactccaccgctgagtacgtaagctaccgtccgagtgacttcggtgcgtatctggatgcgctggaagccgaggttgcacgaggcggtcttattgtgttccacaacggtcacaagtatgacgttcctgcattgaccaaactggcaaagtt                                                        >  1:63124‑M1/1‑80 (MQ=255)`  
                                                                                                                                                                                                                                                    `aGGCTACCCGTGTGGCAAACCAAGCTCAACAGCTTTCCAAATTCTTTGAGTAATCAAACAGGAGAAATCAATATGatcgtttatgacatcgaagctaacgccctcttagagagcgtcactaagttccactgcggggttatctacgactactccaccggtgagtacgtaagctaccgtccgagtgacttcggtacgtatctggatgcgctggaagccgaggttgcacgaggcggtcttattgtgttccacaacggtcacaagtatgacgttcctgcattgaccaaactggcaaagttgcgat                                                   <  1:29306‑M1/301‑227 (MQ=255)`  
                                                                                                                                                                                                                                                             `gtgtGGCAAACCAAGCTCAACAGCTTTCCAAATTCTTTGAGTAATCAAACAGGAGAAACCATTATGatcgtttctgacatcgaagctaacgccctcttagagagcgtcactaagttccactgcggggttatctacgactactccaccgctgagtacgtaagctaccgtccgagtgacttcggtgcgtatctggatgcgctggaagccgaggttgcacgaggcggtcttattgtgttccacaacggtcacaagtatgacgttcctgcattgaccaaactggcaaagttgcaattgaaccgag                                          <  1:311525‑M1/301‑236 (MQ=255)`  
                                                                                                                                                                                                                                                                      `aCCAAGCTCAACAGCTTTCCAAATTCTTTGAGTAATCAAACAGGAGAAACCATTATGatcgtttctgacatcgaagctaacgccctcttagagagcgtcactaagttccactgcggggttatctacgactactccaccgctgagtacgtaagctaccgtccgagtgacttcggtgcgtatctggatgcgctggaagccgaggttgcacgaggcggtcttattgtgttccacaacggtcacaagtatgacgttcctgcattgaccaaactggcaaagttgcaattgaaccgagagttccacc                                 >  1:388105‑M1/1‑57 (MQ=255)`  
                                                                                                                                                                                                                                                                       `acaAGCTAAACAGCTTGCCAAATTCTTTGAGTAATCAAACAGGAGAAACCATTATGatcgtttctgacgtcgaagctaacgccctcttagagagcgtcactaagttccactgcggggttatctacgactactccaccgctgagtacgtaagctaccgtccgagtgacttcggtgcgtatctggatgcgctggaagccgaggttgcacgaggcggtcttattgtgttccacaacggtcacaagtatgacgttcctgcattgaccaaactggcaaagttgcaattgaaccgagagttccacct                                <  1:303663‑M1/300‑246 (MQ=255)`  
                                                                                                                                                                                                                                                                               `aaCAGCTTTCCAAATTCTTTGAGTAATCAAACAGGAGAAACCATTATGatcgtttctgacatcgaagctaacgccctcttagagagcgtcactaagttccactgcgaggttatctacgactactccaccgctgagtacgtaagctaccgtccgagtgacttcggtgcgtatctggatgcgctggaagccgaggttgcacgaggcggtcttattgtgttccacaacggtcacaagtatgacgttcctgcattgaccaaactggcaaagttgcaattgaaccgagagttccaccttcctcgtg                        <  1:345060‑M1/301‑254 (MQ=255)`  
                                                                                                                                                                                                                                                                                      `ttCCAAATTCTTTGAGTAATCAAACAGGAGAAACCATTATGatcgtttctgacatcgaagctaacgccctcttagagagcgtcactaagttccactgcggggttatctacgactactccaccgctgagtacgtaagctaccgtccgagtgacttctgtgcgtatctggatgcgctggaagccgaggttgcacgaggcggtcttattgtgttccacaacggtcacaagtatgacgttcctgcattgaccaaactggcaacgttgcaattgaaccgagagttccaccttcctcgtgagaactg                 >  1:261784‑M1/1‑41 (MQ=255)`  
                                                                                                                                                                                                                                                                                           `aagtagttgagtaaGCAAACAGGAGAAACCCTGATGatcgtgtctgacatcgaagctaacgccctcttagagagcgtcactaagttccactgcggggttatctacgactacgccaccgctgagtacgtaagctaccgtccgagtgacttcggtgcgtatctggatgcgctgtaagccgaggatgcacgagtcggtcttattgtgttccacaacggtcacaagtatgacgttcctgcattgaccaaactggcaaagttgcaattgaaccgagagttccaccttccacgtgagaactatattg            <  1:95504‑M1/295‑266 (MQ=255)`  
                                                                                                                                                                                                                                                                                            `aTTCTTTGAGTAATCAAACAGGAGAAACCATTATGatcgtttctgacatcgaagctaacgccctcttagagagcgtcactaagttccactgcggggttatctacgactactccaccgctgagtacgtaagctaccgtccgagtgacttcggtgcgtatctggatgcgctggaaaccgaggttgcacgaggcggtcttattgtgttccacaacggtcacaagtatgacgttcctgcattgaccaaactggcaaagttgcaattgaaccgagagttccaccttcctcgtgagaactgtattga           <  1:316042‑M1/301‑267 (MQ=255)`  
                                                                                                                                                                                                                                                                                             `ttCTTTGAGTAATCCAACAGGAGAAACCATTATGatcgtttctgacatcgaagctaacgccctcttagagcgcgtcactaagttccactgcggtgttatctacgactactccaccgctgagtacgtaagctaccgtccgagtgacttcggtgcgtatctggatgcgctggaagccgaggttgcacgaggcggtcttattgtgttccacaacggtcacaagtatgacgttcctgcattgaccaaactggcaaagttgcaattgaaccgagagttccaccttcctcgtgagaactgtattgac          <  1:132914‑M1/301‑268 (MQ=255)`  
                                                                                                                                                                                                                                                                                              `tCTTTGAGTAATCAAACAGGAGAAACCATTATGatcgtttctgacatcgaagctaacgccctcttagagagcgtcactaagttccactgcggggttatctacgactactccaccgctgagtacgtaagctaccgtccgagtgacttcggtgcgtatctggatgcgctggaagccgaggttgcacgaggcggtcttattgtgttccacaacggtcacaagtatgacgttcctgcattgaccaaactggcaaagttgcaattgaaccgagagttccaccttcctcgtgagaactgtattgaca         <  1:397303‑M1/301‑269 (MQ=255)`  
                                                                                                                                                                                                                                                                                                     `gTAATCAAACAGGAGAAACCATTATGatcgtttctgacatcgaagctaacgccctcttagagagcgtcactaagttccactgcggggttatctacgactactccaccgctgagtacgtaagctaccgtccgagtgacttcggtgcgtatctggatgcgctggaagccgaggttgcacgaggcggtcttattgtgttccacaacggtcacaagtatgacgttcctgcattgaccaaactggcaaagttgcaattgacccgagagttccaccttcctcgtgagaactgtattgacacccttgt  >  1:410520‑M1/1‑26 (MQ=255)`  

                                                                                                                                                                                                                                                                                                            `|`                                                                                                                                                                                                                                                                                                       
`GGATTCTTGATGACTTTCCAGACGACTACGAGAAGTTTCGCTGGAGAGTCCCATTCTAATACGACTCACTAAAGGAGACACACCATGTTCAAACTGATTAAGAAGTTAGGCCAACTGCTGGTTCGTATGTACAACGTGGAAGCCAAGCGACTGAACGATGAGGCTCGTAAAGAGGCCACACAGTCACGCGCTCTGGCGATTCGCTCCAACGAACTGGCTGACAGTGCATCCACTAAAGTTACCGAGGCTGCCCGTGTGGCAAACCAAGCTCAACAGCTTTCCAAATTCTTTGAGTAATCAAACAGGAGAAACCATTATGTCTAACGTAGCTGAAACTATCCGTCTATCCGATACAGCTGACCAGTGGAACCGTCGAGTCCACATCAACGTTCGCAACGGTAAGGCGACTATGGTTTACCGCTGGAAGGACTCTAAGTCCTCTAAGAATCACACTCAGCGTATGACGTTGACAGATGAGCAAGCACTGCGTCTGGTCAATGCGCTTACCAAAGCTGCCGTGACAGCAATTCATGAAGCTGGTCGCGTCAATGAAGCTATGGCTATCCTCGACAAGATTGATAACTAAGAGTGGTA  >  NC_001604.1/13268‑13861`

  

Alignment Legend

Aligned base mismatch/match (shaded by quality score): `ATCG/ATCG` `< 3 ≤` `ATCG/ATCG` `< 9 ≤` `ATCG/ATCG` `< 20 ≤` `ATCG/ATCG` `< 34 ≤` `ATCG/ATCG` `< 38 ≤` `ATCG/ATCG`

Unaligned base: `atcg`    Masked matching base: `atcg`    Alignment gap: `‑`    Deleted base: `‑`

Reads not counted as support for junction

read\_name Not counted due to insufficient overlap past the breakpoint.

read\_name Not counted due to not crossing MOB target site duplication.
